# Strategic Electrochemical Determination of Nitrate over Polyaniline/Multi-Walled Carbon Nanotubes-Gum Arabic Architecture

**DOI:** 10.3390/nano12193542

**Published:** 2022-10-10

**Authors:** Samia Abdulhammed Mohamad Kosa, Amna Nisar Khan, Sana Ahmed, Mohammad Aslam, Wafa AbuBaker Bawazir, Abdul Hameed, Muhammad Tahir Soomro

**Affiliations:** 1 Department of Chemistry, Faculty of Science, King Abdulaziz University, Jeddah 21589, Saudi Arabia; 2 Centre of Excellence in Environmental Studies (CEES), King Abdulaziz University, Jeddah 21589, Saudi Arabia; 3 Department of Applied Chemistry, Engineering School, Kyungpook National University, Daegu 41566, Korea; 4 National Center of Physics, Quaid-e-Azam University, Islamabad 44000, Pakistan

**Keywords:** polyaniline, multi-walled carbon nanotubes, gum arabic, nitrate determination, voltammetry

## Abstract

Significant agricultural and industrial activities necessitate the regular monitoring of nitrate (NO_3_^−^) ions levels in feed and groundwater. The current comparative study discloses an innovative user-friendly electrochemical approach for the determination of NO_3_^−^ over polyaniline (PAni)-based modified electrodes. The electrochemical sensors concocted with PAni, multi-walled carbon nanotubes (CNT), and gum arabic (GA). The unique electrode material GA@PAni-CNT was synthesized by facile one-pot catalytic polymerization of aniline (Ani) with FeCl_3_/H_2_O_2_ in the presence of CNT and GA as integral components. As revealed by cyclic voltammetry (CV), the anchoring/retention of NO_3_^−^ followed by reduction is proposed to occur when a GA@PAni-CNT electrode is immersed in phosphate buffer electrolyte containing NO_3_^−^ that eventually results in a significantly higher redox activity of the GA@PAni-CNT electrode upon potential scan. The mechanism of NO_3_^−^ anchoring may be associated with the non-redox transition of leucomeraldine salt (LS) into emeraldine salt (ES) and the generation of nitrite (NO_2_^−^) ions. As a result, the oxidation current produced by CV for redox transition of ES ↔ pernigraniline (PN) was ~9 times of that obtained with GA@PAni-CNT electrode and phosphate buffer electrolyte, thus achieving indirect NO_3_^−^ voltammetric determination of the GA@PAni-CNT electrode. The prepared GA@PAni-CNT electrode displayed a higher charge transfer ability as compared to that of PAni-CNT and PAni electrodes. The optimum square wave voltammetric (SWV) response resulted in two linear concentration ranges of 1–10 (R^2^ = 0.9995) and 15–50 µM (R^2^ = 0.9988) with a detection limit of 0.42 µM, which is significantly lower. The GA@PAni-CNT electrode demonstrated the best detection, sensitivity, and performance among the investigated electrodes for indirect voltammetric determination of NO_3_^−^ that portrayed the possibility of utilizing GA—stabilized PAni and CNT nanocomposite materials in additional electrochemical sensing applications.

## 1. Introduction

In recent years, organic semiconductors have been researched for electrochemical determination applications because of their adaptable conducting nature and versatile applications in high-performance electronics [1,2,3]. Among the various organic semiconductors, PAni has been demonstrated to be a very efficient, non-toxic, low-cost electrode material and is especially suitable for electrochemical sensing applications [4,5,6,7]. Still, the mechanism of electrochemical determination of various analytes using PAni as an electrode material has yet to be fully explained [8,9]. The synthesis of PAni is also considered to be relatively easy, with good environmental stability. However, the main disadvantage of PAni is its inferior electrochemical cycling stability [10,11,12]. Moreover, PAni is a redox-active material with three distinguishable oxidation states, leucoemeraldine (fully reduced), emeraldine (half oxidized), and pernigraniline (fully oxidized), which can, in principle, exist as salts or bases. PAni’s two redox pairs, LS ↔ ES and ES ↔ PN oxidation/reduction reactions, are positioned at the potentials roughly higher than + 0.3 V and lower than + 0.1 V, respectively [13,14,15,16]. However, the significance of PAni’s redox reactions in electrochemical sensing applications is not described or well understood.

Substantial work has been performed to ease this limitation. Currently, CNTs are the predominant nanomaterial used to enhance electrochemical stability/cyclability of PAni [5,17]. CNTs are described as scaffolds made entirely of sp^2^ carbon atoms with a diameter in the nanoscale range and are generally divided into single-walled carbon nanotubes and multi-walled carbon nanotubes based on their structure. The excellent electrocatalytic activity of CNT is also well documented over the years, as well as CNTs’ high specific surface area, high conductivity, adsorption capability, and extraordinary optical and mechanical properties [18,19]. Nanocomposites of PAni and CNT have been developed for a variety of applications including lithium ion batteries, diodes, solar cells, supercapacitors, photocatalysts, gas sensors, and chemical/biosensors [5,20]. Thus, in recent decades, many researchers have investigated the voltammetric determination of various organic molecules using PAni-CNT nanocomposite as an electrode material. A considerable study suggests that the presence of CNTs’ surface oxygen functionalities is likely to affect the electrochemical behavior of the resulting PAni-CNT-based electrodes [21,22]. Consequently, the adsorption/immobilization of some of the analytes on the PAni-CNT nanocomposite electrode has been reported [23]. However, to the best of our knowledge, PAni-CNT nanocomposites have not been applied for the electrochemical determination of NO_3_^−^.

In an optimistic view, the combination of PAni and CNT may produce a unique material with improved electrochemical activity and good processability. However, the application of PAni-CNT-based electrodes is directly limited by the stabilization of the electrode material in aqueous media [24,25]. To achieve this, the resulting PAni-CNT nanocomposite is functionalized with natural polymer GA. GA is a mixture of water-soluble polysaccharides exuded from various species of acacia trees [26]. Among the different polymers, GA has found a variety of applications, especially in industry as an emulsifier and stabilizer [27]. The primary goal of GA is to bind PAni and CNT components together and glue the nanocomposite onto the electrode surface so that nanocomposite will not leach into the aqueous medium [28,29]. Due to the presence of many ionizable groups from the monosaccharides moieties, especially uronic acid, and proteinaceous groups, GA usually behaves as a polyelectrolyte in the aqueous medium that is positively or negatively charged depending on the pH of the medium [30]. Some recent studies have shown the feasibility of using GA as a corrosion inhibitor and stabilizer of inorganic particles [31]. However, GA-stabilized electrode material still finds limited use in electrochemical sensing applications. Hence, a proper study of GA-stabilized electrode material is required to fully elucidate its electrochemical stability and boost interest for further use in additional electrochemical sensing applications.

The contamination of NO_3_^−^ ions is ubiquitous within environmental, food, natural water, and physiological systems due to its use as fertilizer in agriculture and from the oxidation of nitrogenous waste products [32,33]. High NO_3_^−^ concentration in drinking water could lead to an increased risk of methemoglobinemia in infants and stomach cancer in adults [34]. Over the last few decades, the problem has been widely accepted, and, as a consequence, substantial work has been performed aimed at controlling NO_3_^−^ levels within a wider environment and food products [35]. Most of the methods for NO_3_^−^ determination have been based on spectrophotometric and chromatographic techniques [36]. However, the main disadvantages of these methods are their laborious and time-consuming preparation steps. Therefore, electrochemical methods are the most convenient alternatives for the rapid and simple determination of NO_3_^−^ [37]. The direct determination of NO_3_^−^ on a bare unmodified electrode is limited because of the slow kinetics of the charge transfer and surface passivation effect. Therefore, the chemically modified electrode with an easy surface regeneration ability represents a more suitable choice for the electrochemical determination of NO_3_^−^ [38,39]. However, the direct reduction of NO_3_^−^ on such chemically modified electrodes also affected their activity, which decreased very quickly after the first scan [34,39,40,41]. A better choice for the NO_3_^−^ determination is, therefore, based on the indirect determination of the chemically modified electrode. While our understanding of the indirect electrochemical determination of NO_3_^−^ has increased [32,42], a substantial degree of hesitation still remains mainly because of the somewhat unknown mechanism of determination. Therefore, the inherent complexities in the indirect electrochemical determination of NO_3_^−^ using chemically modified electrodes limit their widespread applicability.

No research studies have clearly elaborated the indirect electrochemical determination of NO_3_^−^ on chemically modified electrodes, especially those that are PAni based; therefore, in this work, a viable electrochemical approach for the indirect determination of NO_3_^−^ based on GA@PAni-CNT nanocomposite modified electrode is presented. The GA@PAni-CNT nanocomposite was prepared by the one-pot catalytic polymerization of aniline with FeCl_3_/H_2_O_2_ in the presence of co-partner CNT and GA. The GA@PAni-CNT electrode was prepared by fabricating a glassy carbon electrode (GCE). The CV oxidation/reduction behavior of GA@PAni-CNT electrode was investigated in phosphate buffer electrolyte with and without NO_3_^−^. The stability and reusability of the GA@PAni-CNT electrode were studied. The optimum SWV conditions for NO_3_^−^ determination with the GA@PAni-CNT electrode were acquired. The quantitative relationships for the lowest detection of NO_3_^−^ on GA@PAni-CNT were established. Finally, the NO_3_^−^ determination mechanism of the GA@PAni-CNT electrode was construed in detail.

## 2. Experimental

### 2.1. Chemicals

Aniline (C_6_H_7_N, 99%, Ani), iron (III) chloride hexahydrate (FeCl_3_·6H_2_O), hydrogen peroxide (H_2_O_2,_ 30%), hydrogen chloride (HCl, 37%), sodium nitrate (NaNO_3_), phosphoric acid (H_3_PO_4_), acetic acid (CH_3_COOH), boric acid (H_3_BO_3_), sodium hydroxide (NaOH), potassium ferricyanide K_3_[Fe(CN)_6_], 5% solution of Nafion^®^, and dimethylformamide (DMF) were chemicals of high purity, and were purchased from Sigma-Aldrich, Taufkirchen, Germany. Edible GA was purchased from the local market in Jeddah, Saudi Arabia. CNTs were obtained from the Advanced Carbon Division, Institute of Metal Research, Chinese Academy of Sciences (Shenyang, China) and used as received. All solutions were prepared using Milli-Q water unless stated otherwise. Acetate. 0.1 M pH 5.0 phosphate buffer, and Britton-Robinson buffer were used as the supporting electrolytes. Buffer solutions of different pH range from 3.0 to 8.0 were also prepared. The 0.1 M NaNO_3_ stock solution was prepared fresh every week and kept at room temperature when not in use. The working solutions of NaNO_3_ were prepared with 0.1 M pH 5 phosphate buffer electrolyte. The 5% Nafion^®^ was diluted in DMF to prepare a 0.1% Nafion^®^ solution.

### 2.2. Synthesis of GA@PAni-CNT Nanocomposite

Synthesis of the GA@PAni-CNT nanocomposite was carried out using the catalytic polymerization of Ani [43]. Measured volume of Ani (0.92 mL, 50 mmol) was stirred in 100 mL of 1 M HCl for 30 min, followed by addition of FeCl_3_ (16 mg), CNT (8 mg), and GA (0.02%, 5 mg). The mixture was kept under stirring for another 30 min. The polymerization was initiated by the dropwise addition of 0.01 M H_2_O_2_, which was prepared in 50 mL of 1 M HCl. The mixture stirred continuously until the color changed from pale yellow to green. The formed nanocomposite was isolated by centrifugation and washed several times with water to maintain a neutral pH range (6–7). The obtained precipitates were then placed in the vacuum oven at 100 °C for overnight which resulted in black powder. The PAni and PAni-CNT nanocomposite was synthesized accordingly, where in the former polymerization was performed without CNT and GA, and in the latter polymerization was performed without GA only.

### 2.3. Characterization Details

The UV–visible absorption spectra of the synthesized PAni, PAni-CNT, and GA@PAni-CNT nanocomposites were acquired by a UV–visible spectrophotometer (Cary 60, Agilent Technology, Santa Clara, CA, USA) in DMF. Chemical structural differences between PAni, PAni-CNT, and GA@PAni-CNT nanocomposite were analyzed using a DXR Raman Microscope (Thermo Scientific, Waltham, MA, USA) equipped with an excitation source diode-pumped solid-state (DPSS) 532 nm laser at 8 mW power and a Cary 630 Fourier-transform infrared (FTIR) spectrophotometer (Agilent Technology, Santa Clara, CA, USA). For phase identification analysis, an automated multipurpose X-ray diffractometer (Ultima IV, Rigaku, Tokyo, Japan) equipped with a Cu Kα radiation source in the 2θ range of 10° to 80° at 40 kV accelerating voltage and a current of 40 mV was used. Nitrogen species of GA@PAni-CNT nanocomposite were distinguished using an X-ray photoelectron spectrometer (PHI 5000 Versa Probe II, ULVAC-PHI Inc., Chigasaki, Japan) in the binding energy range of 0 eV to 1350 eV. The morphological features such as shape, size, porosity, and topography of PAni, PAni-CNT, and GA@PAni-CNT nanocomposites were characterized using a field emission electron microscope (FESEM, JEOL, JSM-7600F, Tokyo, Japan).

### 2.4. Preparation of GA@PAni-CNT Electrode

Before the commencement of each experiment, the GCE surface was polished with alumina and diamond slurry to a mirror finish, followed by sonication for about 1 min in 10% nitric acid, ethanol, and double deionized water, and finally rinsed with double deionized water, and dried in air. The modification of GA@PAni-CNT on GCE was performed as follows: 1 mg of GA@PAni-CNT nanocomposite was suspended in 1 mL of DMF and subjected to sonication for 15 min to obtain a homogenous suspension. A polished GCE was coated with 5 µL of the prepared nanocomposite suspension to obtain the GA@PAni-CNT electrode. To avoid leaching of GA@PAni-CNT from the GCE surface into the test solution, a protecting layer of Nafion^®^ was cast on the GA@PAni-CNT electrode by spreading a 5 µL 0.1% Nafion^®^ solution. GCEs modified with PAni and PAni-CNT were also prepared for comparison purposes.

### 2.5. Electrochemical Studies

Electrochemical impedance spectroscopy (EIS), CV, and SWV measurements were performed on a VSP-300 multichannel potentiostat (Biologic Science Instruments, France) based on a three-electrode configuration. The working electrode was a 3 mm GCE, whereas the reference and counter electrodes were saturated calomel electrode (SCE) and Pt wires, respectively. Data acquisition and analysis were performed with EC-Lab software. The EIS experiment was prepared by immersing modified electrode(s) in 0.1 M KCl and 2 mM [Fe(CN)_6_]^3−/4−^ and employing a forward bias of +0.235 V. Then, EIS spectra were generated by scanning the AC frequency between 0.1 Hz and 100 kHz at an AC amplitude of 10 mV. The EIS Nyquist plots were constructed using Randle’s equivalent circuit. The CV curves were recorded between −0.6 and +1.0 V at a scan rate of 100 mV/s in a phosphate buffer electrolyte of 0.1 M and pH 5 containing 40 µM NO_3_^−^. The SWV curves were acquired using a pulse height of 50 mV, a pulse width of 50 ms, a pulse height of 5 mV, and a delay time of 10 s in phosphate buffer electrolyte solutions of varying pH. An inert atmosphere of nitrogen was maintained over the test solution in the cell.

### 2.6. Practical Application

NO_3_^−^ contamination in groundwater is an important source of NO_3_^−^ leaching into the water supply system; therefore, NO_3_^−^ presence was detected in tap water (Jeddah, Saudi Arabia). The sample was filtered using a 0.45 µM membrane filter and refrigerated at 4 °C. For recording the SWV curve, the sample was diluted with 0.1 M pH 3 phosphate buffer electrolyte. Later, SWV curves of the same sample were recorded with different concentrations of NO_3_^−^.

## 3. Results and Discussion

### 3.1. UV–Visible Spectra

**Figure 1:**
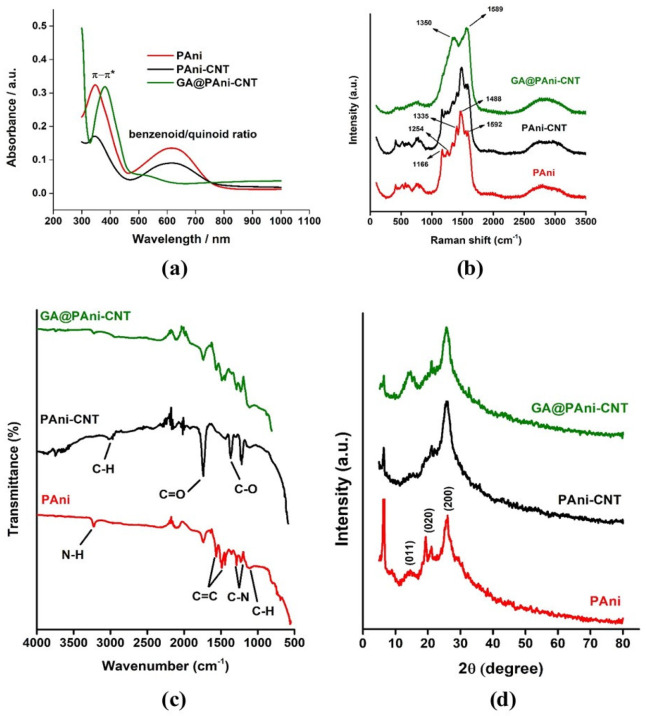
(**a**) UV–visible, (**b**) Raman, (**c**) FTIR, and (**d**) XRD spectra of PAni, PAni-CNT, and GA@PAni-CNT.

As presented in Figure 1a, the oxidation state of PAni in GA@PAni-CNT was investigated by the comparative analysis of UV–visible spectra of PAni, PAni-CNT, and GA@PAni-CNT powders. The characteristic absorption bands typical of PAni were observed at 347 and ~615 nm, respectively. The observations were consistent with that mentioned in the literature [43,44,45]. The band at ~347 nm (UV-region) originated from the π → π* transition in the benzene rings, whereas the band originating from the variation in the benzenoid/quinoid ratio was observed at ~615 nm (visible region). As a rule of thumb, the intensity of the band at ~615 nm, i.e., the benzenoid/quinoid ratio, was employed to estimate the degree of oxidation of PAni. The lower benzenoid/quinoid ratio resulted in a higher intensity of the ~615 nm band and indicated the half-oxidized emeraldine form of PAni. It was anticipated that oxygen functional groups such as the carboxylic groups of CNT have interactions with PAni. However, contrary to some reports [46,47], the benzenoid/quinoid ratio does not reveal a significant change occurring to the PAni backbone due to the fact that the interactions between the quinoid ring of PAni and CNT were somewhat weaker than expected. The UV–visible spectrum of PAni-CNT presents two absorption bands similar to those obtained with pure PAni, more or less at the same wavelengths, indicating the presence of the emeraldine form of PAni-CNT. On the other hand, a mild decrease in the benzenoid–quinoid transition implied a lower conductivity of PAni-CNT in comparison to pure PAni. In the GA@PAni-CNT spectrum, only the transition due to the benzenoid ring is present at 383 nm, whereas the band typical of quinoid transition at ~615 nm completely disappeared [48], which indicated the complete reduction of emeraldine into a leucoemeraldine form. Thus, the redshift of the benzenoid band is the result of the increased conjugation of PAni. Therefore, the UV–visible spectra provided sufficient evidence of fully reduced leucoemeraldine formation upon stabilizing PAni-CNT with GA.

### 3.2. Raman and FTIR Analysis

Raman and FTIR spectroscopy were employed to confirm the leucoemeraldine form of PAni in the GA@PAni-CNT nanocomposite. The Raman spectra of PAni, PAni-CNT, and GA@PAni-CNT are compared in Figure 1b. From the spectra, the in-plane C-H contribution of quinoid and benzenoid rings, the C-N stretching vibration, and the C=C stretching vibrations of quinoid and benzene rings for both PAni and PAni-CNT are clearly seen at wavenumbers ~1166, ~1254, ~1335, ~1488, and ~1592 cm^−1^, respectively [49,50]. Consistent with the UV–visible spectroscopy results, the mild blueshift of the quinoid stretching vibration of PAni-CNT was due to the weaker interactions between PAni and CNT, indicating a smaller charge carrier ability of PAni-CNT than that of pure PAni [51,52]. Notably, the original complex spectrum of PAni-CNT was reduced to two dominating peaks at 1350 and 1589 cm^−1^ when GA was blended with PAni-CNT. These two peaks were very similar to the D and G bands of CNT reported in the literature [51] and corresponded to C-N and C=C stretching vibrations of the reduced PAni form. Thus, Raman spectroscopy clearly indicated the formation of the leucoemeraldine form of PAni in the GA@PAni-CNT nanocomposite.

The FTIR spectra of PAni, PAni-CNT, and GA@PAni-CNT are presented in Figure 1c. For pure PAni, the absorption peaks at ~1562 and ~1472 cm^−1^ belong to the C=C stretching vibrations of the quinoid and benzenoid rings, respectively, whereas the N-H stretching band of amine appears at ~3227 cm^−1^. The peaks at ~1280, ~1240, and ~1144 cm^−1^ are characteristic of C-N stretching of the secondary amide group and C-H bending vibrations of a benzene ring, which confirms the emeraldine form of PAni [47,53,54]. However, in the PAni-CNT spectrum, PAni FTIR features are suppressed by the prominent carboxylic group C=O and C-O stretching vibration at ~1747 cm^−1^, ~1369, and ~1208 cm^−1^. In addition, symmetric and asymmetric C-H stretches of PAni-CNT are observable in 2978–2841 cm^−1^ region. The FTIR spectrum of GA-treated PAni-CNT was similar to pure PAni and showed a significant reduction in number of bands that might be attributed to GA covering PAni-CNT [55,56]. Nevertheless, the FTIR spectrum of GA@PAni-CNT further confirmed the formation of the leucoemeraldine form of PAni in GA@PAni-CNT, as the intensity of quinoid C=C stretching was identical to the benzenoid C=C stretching.

### 3.3. XRD Pattern

The X-ray diffraction (XRD) patterns of the synthesized PAni, PAni-CNT, and GA@PAni-CNT are shown in Figure 1d. The pure PAni exhibited typical reflections at 2θ = ~14.65°, ~19.33°, and ~25.98° that corresponded to the (011), (020), and (200) crystalline planes of PAni in its emeraldine form, respectively. The observed values were in good agreement with those reported in the literature [47]. The PAni-CNT displayed an intense reflection at 2θ = ~25.79° due to the overlap of the CNT diffraction peak with that of PAni and implied an improved crystalline nature of the PAni-CNT nanocomposite. The other low intensity reflections may be attributed to the repeating units of PAni and weaker interactions between PAni and CNT [57]. For GA@PAni-CNT, the characteristic reflections were identical to the XRD pattern of pure PAni; however, the broadening of the peaks indicated the transformation of emeraldine into the leucoemeraldine form due to GA free reducing groups [58]. Moreover, no distinct reflection of GA was noticed, as GA is amorphous in nature. The broadening of the peak at 2θ = 14.65° suggested cross-linking between PAni and GA. A careful comparison of the XRD results revealed an interaction of PAni-CNT and GA with enhanced surface coverage that not only suppressed the characteristic reflections arising from the base material but also caused the comparative broadening.

### 3.4. XPS Study

**Figure 2:**
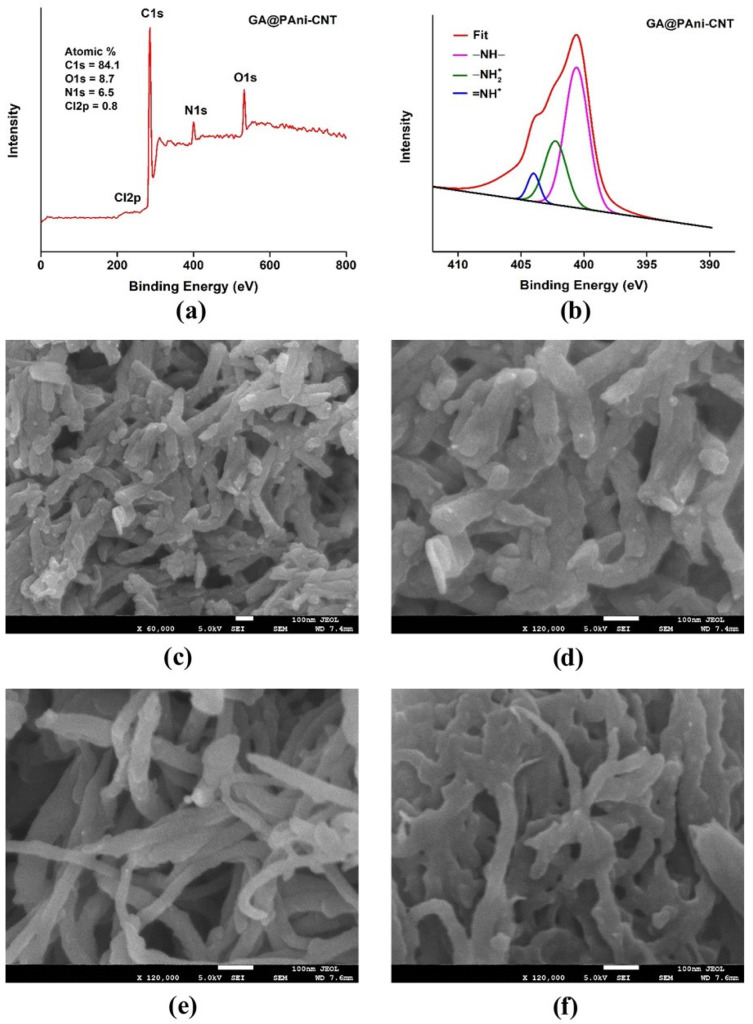
(**a**) XPS spectrum, (**b**) N1s deconvoluted analysis, and FESEM images of PAni (**c**,**d**), PAni-CNT (**e**), and GA@PAni-CNT (**f**).

The leucoemeraldine form of PAni in the GA@PAni-CNT nanocomposite was further investigated by X-ray photoelectron spectroscopy (XPS). In Figure 2a,b, the survey scan and N1s spectra of GA@PAni-CNT are presented. The presences of C (84.1%), O (8.7%), N (6.5%), and Cl (0.8%) were confirmed, as expected, whereas the deconvolution of N1s high-resolution spectrum revealed three embedded peaks at 400.50, 402.30, and 403.85 eV, which were associated with benzenoid amine (−NH−), protonated amine (−NH_2_^+^), and protonated imine (=NH^+^), respectively [59,60,61,62,63]. It must be emphasized here that although the values are slightly higher, they were in accordance with those reported in the NIST database [64]. No traces of quinoid imine (=N−) were identified. It appeared that PAni in the GA@PAni-CNT nanocomposite was in its fully reduced leucoemeraldine form with a small percentage of protonated imine, which is consistent with previous characterizations of the nanocomposite. Thus, by blending PAni-CNT with GA, the leucoemeraldine form of PAni in GA@PAni-CNT is achieved.

### 3.5. FESEM Surface Morphology

The surface morphologies of PAni, PAni-CNT, and GA@PAni-CNT were examined by FESEM analysis and the images at various resolutions are presented in Figure 2c–f. The images of PAni at magnifications of 60,000× and 120,000× revealed the short and random quasi-dimensional nanofibers with a typical dimension of tens to hundreds of nanometers [65,66]. The images of PAni-CNT at a magnification of 120,000× exhibited a burr-like uniform coating of PAni at the surface of nanotubes. The CNTs’ surface oxygen functional groups and defects lead to the attachment/adsorption of PAni with CNT. In addition, π–π stacking interactions and hydrogen bonding were also helpful in wrapping PAni around the CNTs, creating a structure of PAni-CNT that is represented as coaxial cylinders [67]. The presence of GA in PAni-CNT has a significant effect on the overall dispersibility and porosity of the resulting GA@PAni-CNT nanocomposite [68,69]. The nanocomposite seemed more dense, smooth, and mesoporous than PAni-CNT, which was valuable from an electrochemical determination point of view. The hydration effects of GA allow uniform coating of GA as a thin film on PAni-CNT, which is observable in the FESEM images of GA@PAni-CNT at 120,000×.

### 3.6. EIS Performance

**Figure 3:**
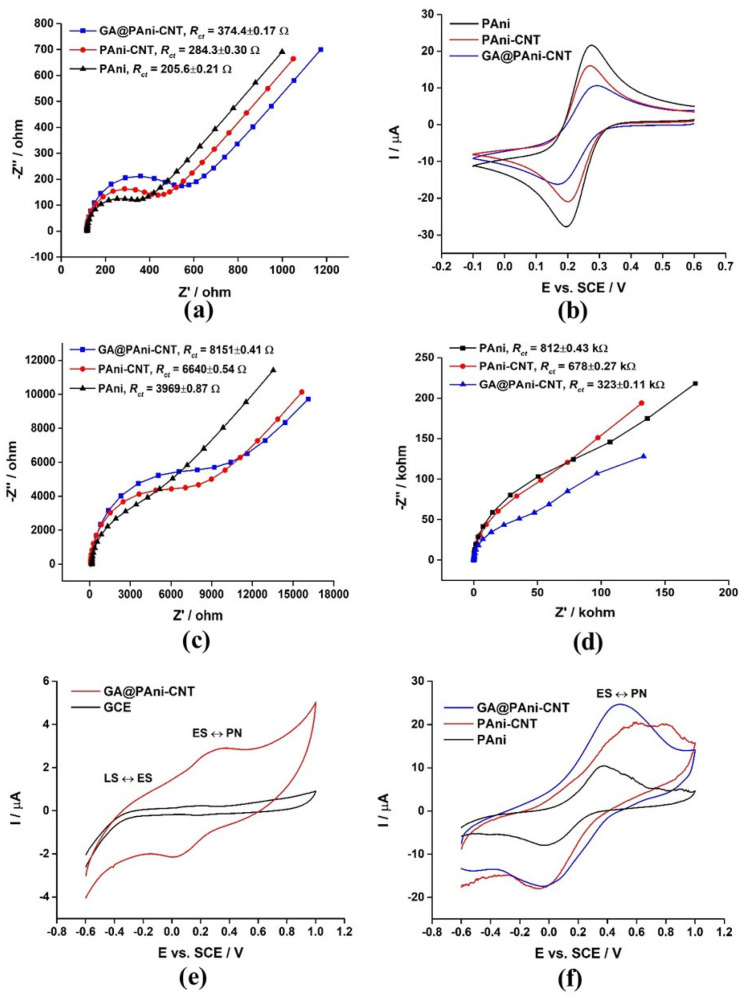
(**a**) EIS spectra and CV curves of PAni, PAni-CNT, and GA@PAni-CNT against 2 mM [Fe(CN)_6_]^3−/4−^ in 0.1 M KCl (**a**,**b**), EIS spectra of the same in (**c**) 0.1 M pH 7 phosphate buffer and (**d**) 0.1 M pH 5 phosphate buffer containing 40 µM NO_3_^−^, (**e**) CV curves of bare GCE in 0.1 M pH 5 phosphate containing 40 µM NO_3_^−^ and GA@PAni-CNT in 0.1 M pH 5 phosphate buffer, and (**f**) CV comparison in 0.1 M pH 5 phosphate containing 40 µM NO_3_^−^.

The electrode preparation steps are already explained in the experimental section. The EIS performance, which is required to validate the high charge transfer ability of the prepared electrode(s), is, therefore, evaluated [70,71,72]. The resulting EIS spectra of PAni, PAni-CNT, and GA@PAni-CNT against standard redox probes [Fe(CN)_6_]^3−/4−^ (ferricyanide/ferrocyanide couple) are illustrated in Figure 3a. In EIS, a small semicircle at low frequency would imply a lower charge transfer resistance (*R_ct_*) and vice versa. Apparently, GA@PAni-CNT showed a larger arc than PAni and PAni-CNT, indicating the lower conductivity of the prepared electrode. The *R_ct_* values of PAni, PAni-CNT, and GA@PAni-CNT at +0.235 V (formal potential of [Fe(CN)_6_]^3−/4−^ vs. SCE) were ~205.6 ± 0.21, ~284.3 ± 0.30, and ~374.4 ± 0.17 Ω, respectively, which revealed the hindered charge transfer of GA@PAni-CNT, followed by PAni-CNT and PAni. This observation was in agreement with the fact that LS is an electrically non-conducting form of PAni with no double bonds between the benzene rings and benzenoid amine. However, in the case of ES, which is a protonated form of emeraldine base in an acidic medium, radical cations of nitrogen atoms (positive polarons) work efficiently as charge carriers to transfer electrical current between benzene rings. The transformation in PAni and PAni-CNT electrodes was identified as the source of the lower charge transfer resistance, as in both of the electrodes PAni exists in its emeraldine form. Moreover, the lower conductivity of PAni-CNT in comparison to that of PAni was favorable, because the oxygen functional groups of CNT absorb radical cations to reduce their content of ES given that the conductivity depends on both the content and mobility of radical cations [73,74,75,76]. The corresponding CV curves of PAni, PAni-CNT, and GA@PAni-CNT reflect the same observation as in EIS, represented in Figure 3b, where the well-defined oxidation/reduction peaks are attributed to the reversible electrode reaction of the [Fe(CN)_6_]^3−/4−^ redox couple. The observed CV currents are in the following order: PAni > PAni-CNT > GA@PAni-CNT. Hence, both EIS and CV demonstrate that the GA@PAni-CNT electrode has the leucoemeraldine form of PAni as a result of the blending of GA with PAni-CNT.

As the current flow at open circuit voltage (*E_oc_*) and zero-current potential might provide further insight regarding the behavior of the prepared electrodes in natural state, the EIS spectra of PAni, PAni-CNT, and GA@PAni-CNT were recorded in 0.1 M pH 7 phosphate buffer electrolyte using the same frequency range and amplitude and are presented in Figure 3c. The results were consistent with the EIS spectra (Figure 3a), suggesting the dependence of the EIS performance of the prepared electrodes on the presence of the leucoemeraldine or emeraldine form of PAni in the composite material. On the contrary, when EIS measurements were performed in 0.1 M pH 5 phosphate buffer electrolyte with NO_3_^−^ as an analyte, the charge transfer order of the three electrodes was reversed as compared to the order observed earlier with either the 0.1 M KCl and 2 mM [Fe(CN)_6_]^3-/4-^ or 0.1 M pH 7 phosphate buffer electrolytes (Figure 3d). Accordingly, the new order of charge transfer resistance appeared as PAni > PAni-CNT > GA@PAni-CNT, which demonstrated that in the presence of NO_3_^−^ in phosphate buffer electrolyte, the GA@PAni-CNT exhibited the highest charge transfer ability, followed by PAni-CNT and PAni.

**Scheme 1:**
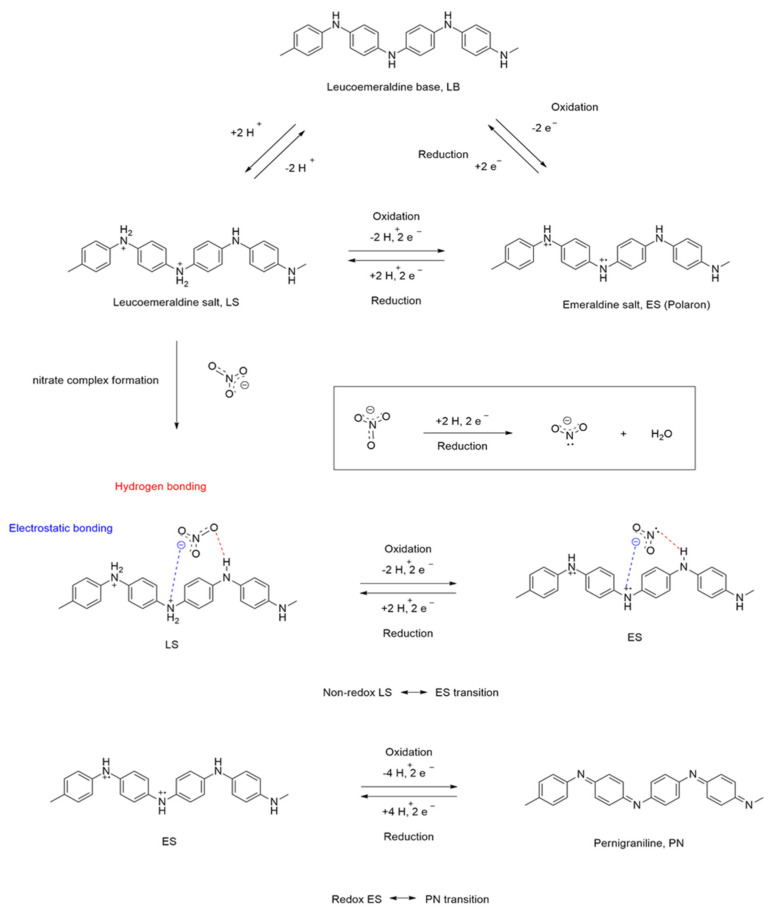
Schematic representation of nitrate complex formation with LS, subsequent non-redox LS ↔ ES transition, and redox ES ↔ PS transition.

Based on PAni’s non-redox protonation and redox oxidation mechanism, the explanation of the high conductivity of GA@PAni-CNT electrode in NO_3_^−^ containing phosphate buffer electrolyte is as follows: PAni in its conductive form is a unique material; however, it cannot be converted to its conductive form without redox oxidation [13,76,77]. While in the acidic electrolyte, proton attachment on (-NH-) turns the leucoemeraldine base to LS (Scheme 1), which is a non-redox doping process. The conductivity of PAni also changes depending on the anion of the acid or added during the protonation of the emeraldine base. Upon NO_3_^−^ addition in the same acidic electrolyte solution, NO_3_^−^ complex formation occurred through hydrogen and electrostatic attractions at the (-NH-) and (-NH_2_^+^) sites, as reported in the literature [78,79,80,81]. It is worthwhile to point out that after NO_3_^−^ complex formation in the absence of applied potential, NO_3_^−^ reduction into NO_2_^−^ by LS on GA@PAni-CNT appeared feasible [78,80]. As a result, radical cations formed at the (-NH-) were believed to be responsible for the significantly lower charge transfer resistance of GA@PAni-CNT than those of PAni-CNT and PAni in 0.1 M pH 5 phosphate buffer electrolyte containing µM NO_3_^−^. On the other hand, NO_3_^−^ complex formation with ES seems thermodynamically unfavorable. The number of radical cations within the polymer, the high mobility of the radical cation, and the electrostatic repulsion between NO_3_^−^ and unpaired electron do not allow nitrate to coordinate with ES. The higher the content of the ES unit, the lesser the attraction between the electrode surface and NO_3_^−^. As a result, the ES content of the PAni electrode remained unchanged, as well as the charge transfer resistance. However, for PAni-CNT, due to the lesser content of ES units in the nanocomposite, conversion of some LS units into ES is possible. Hence, an increase in the conductivity of PAni-CNT electrode in comparison to that of PAni occurred. However, the maximum conductivity was observed when electrode had a higher content/unit of the fully reduced leucoemeraldine form, as in the case of the GA@PAni-CNT electrode. Moreover, during the course of the redox transition of ES ↔ PN, the oxidation of NO_2_^−^ into NO_3_^−^ by applied potential is plausible, which cannot be electrochemically reduced into NO_2_^−^ again as electrochemical reduction of NO_3_^−^ occurs at a sufficiently negative potential, which is indicative of the fact that the redox transition of ES ↔ PN caused regeneration of NO_3_^−^.

### 3.7. CV Behavior

Taking into account the non-redox transition of LS ↔ ES in the presence of NO_3_^−^, the mechanism of indirect electrochemical determination of NO_3_^−^ was extracted by virtue of the CV behavior of PAni, PAni-CNT, and GA@PAni-CNT in 0.1 M pH 5 phosphate buffer electrolyte within a stable potential window of −0.6 to +1.0 V. It can be explained that the maximum CV current is exhibited when the number of radical cations on the electrode surface is maximized. As a result, the maximum conversion of LS into ES, which ultimately produces high CV current, is directly dependent on the concentration of NO_3_^−^ in the phosphate buffer electrolyte. Hence, by measuring the electrochemical oxidation current of ES into fully oxidized form PN, indirect electrochemical determination of NO_3_^−^ is possible. Figure 3e shows the CV curve for bare GCE taken at 100 mV/s in 0.1 M pH 5 phosphate buffer electrolyte and 40 µM NO_3_^−^, where no clear oxidation/reduction peaks were visible, indicating that GCE is a non-catalytic electrode material for the determination of NO_3_^−^ [34,82]. As a result, in the GA@PAni-CNT electrode CV curve at 100 mV/s in 0.1 M pH 5 phosphate buffer electrolyte, one oxidation peak at ~−0.16 V due to the transition of LS ↔ ES and one redox pair positioned at ~0.32/0.039 corresponding to the ES ↔ PN transition was recognizable. Interestingly, the potentials of the GA@PAni-CNT redox peaks were shifted toward lower values with respect to those reported for pure PAni [13,78,79], which was appreciable in terms of easier and faster detection of the targeted analyte. On the other hand, the current density of the ES ↔ PN transition was more pronounced than that of the LS ↔ ES transition, reflecting that the second redox activity can be utilized for electrochemical determination applications. In other words, the redox activity of the LS ↔ ES transition in the NO_3_^−^ solution was suppressed. These differences were the consequences of the incorporation of CNT and GA into PAni and the appearance of two redox activities is indicative of the leucoemeraldine form of PAni in the GA@PAni-CNT nanocomposite.

The redox behavior of the PAni electrode at 100 mV/s in 0.1 M pH 5 phosphate buffer electrolyte and 40 µM NO_3_^−^ is presented in Figure 3f. The ES ↔ PN redox transition is noticeable at ~0.379/−0.037, while the peak currents are slightly higher than the one observed with GA@PAni-CNT in 0.1 M pH 5 phosphate buffer electrolyte. It is worthwhile to mention here that the CV currents of the PAni electrode is not dependent on the presence of NO_3_^−^ ions in the buffer medium. As already discussed, the PAni electrode redox activity is exclusively due to the redox transition of ES ↔ PN. Furthermore, the deterioration of the PAni film electrode as the potential became progressively more positive was evident from the CV curve, which might be the reason for combining PAni with CNT. The redox activity of the PAni-CNT electrode in 0.1 M pH 5 phosphate buffer electrolyte and 40 µM NO_3_^−^ solution relative to the PAni electrode is significantly improved. It is assumed that due to the slightly lower content of the emeraldine form in the PAni-CNT electrode, the conversion of some leucoemeraldine form by NO_3_^−^ to the emeraldine form is likely. As a result, more ES is present to oxidize and consequently, more redox activity is observed. The differences in CV behavior of PAni and PAni-CNT are certainly due to the involvement of NO_3_^−^ in the non-redox conversion of leucoemeraldine to emeraldine as suggested in Scheme 1. The stability of the PAni-CNT film electrode was questionable, as the film deteriorated with the increase in the potential. The issue was resolved by incorporating GA as a stabilizer/binder in the PAni-CNT nanocomposite matrix, because the same is known to stabilize the nanocomposite and prevents electrode passivation [31,83,84]. Consequently, the smooth and further improved CV curve was observed with GA@PAni-CNT, whereas the amount of current generated by GA@PAni-CNT in 0.1 M pH 5 phosphate buffer electrolyte and 40 µM NO_3_^−^ was ~9 times higher than produced in 0.1 M pH 5 phosphate buffer electrolyte. Therefore, the non-redox transition of LS ↔ ES in the presence of NO_3_^−^ offers a great advantage for indirect determination of NO_3_^−^ by observing the redox transition of ES ↔ PN. Additionally, the minor reduction peak at ~−0.53 V was assigned to the transition of LS ↔ ES.

### 3.8. GA@PAni-CNT as a Suitable Electrode

**Figure 4:**
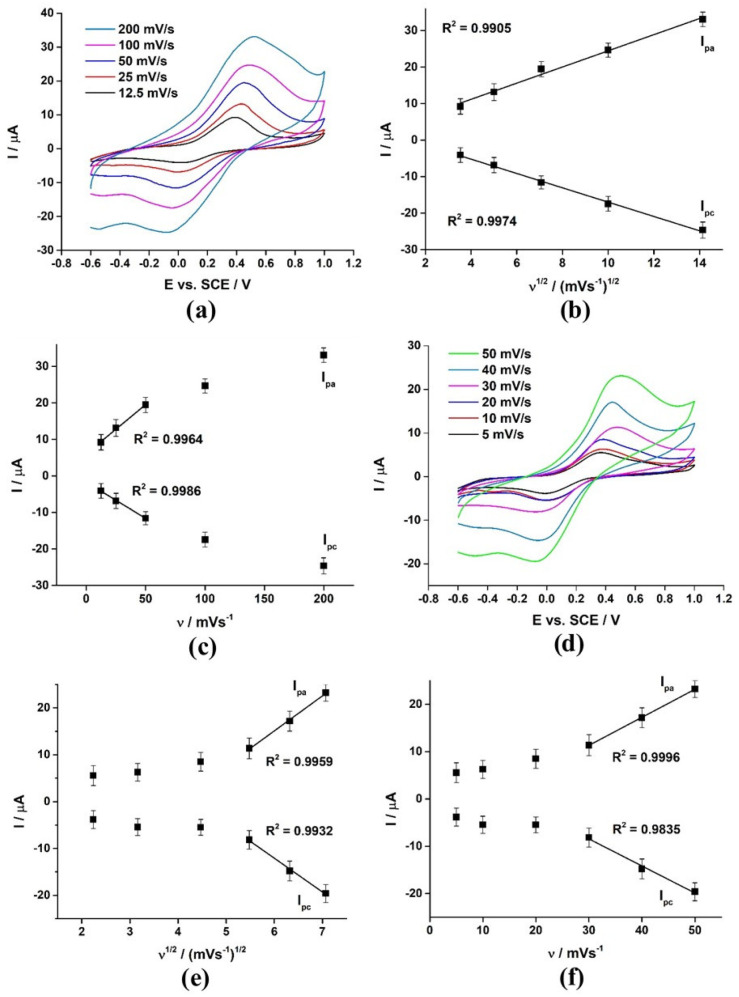
(**a**,**d**) CV curves at different scan rate ranges, (**b**,**e**) *I* vs. *v* plots, and (**c**,**f**) *I* vs. *v*^1/2^ plots.

It is reported that oxidation of PAni leads to the passivation of the electrode due to the electrodeposition of PAni on the electrode surface. The passivated electrode may no longer be effective in repeated studies without brushing off the deposited layer for each scan. A clear picture of the redox transition of ES ↔ PN, either limited by diffusion or adsorption, was therefore necessary. The CV curves of GA@PAni-CNT electrode measured at different scan rates using 0.1 M pH 5 phosphate buffer electrolyte and 40 µM NO_3_^−^ in the potential window of −0.6 V and +1.0 V are depicted in Figure 4a. However, the increase in current with the increase in scan rate, the broadening and distancing of anodic and cathodic peaks revealed the quasi-reversible nature of ES ↔ PN transitions. The gradual increase in the redox peak currents as a function of scan rate suggests the highly rated performance of the GA@PAni-CNT electrode, whereas the shifts in the redox peaks were due to the increased electrode resistance. In Figure 4b, the redox peak currents increased linearly with the square root of the scan rate. The correlation coefficients (*R*^2^), which can be ascribed to the linearity of the process, were 0.9905 and 0.9974 for anodic and cathodic peaks, respectively. The data suggested the presence of a diffusion-controlled ES ↔ PN redox transition within the scan rate range of 12.5 mV/s–200 mV/s. On the other hand, the non-linearity of the redox peak currents with scan rate, as seen in Figure 4c, indicated that the ES ↔ PN redox transition is also controlled by the adsorption mechanism in the scan rate range of 12.5–50 mV/s. Further study through narrowing the scan range (Figure 4d–f) showed the non-linearity of the redox peaks currents with both the scan rate and square root of the scan rate, revealing that the ES ↔ PN redox transition in the scan rate range of 30–50 is controlled by both diffusion and adsorption mechanisms. Therefore, faster scan rate techniques were more suitable to utilize the redox activity of the GA@PAni-CNT electrode for the electrochemical determination application without observing the passivation of the electrode surface.

**Figure 5:**
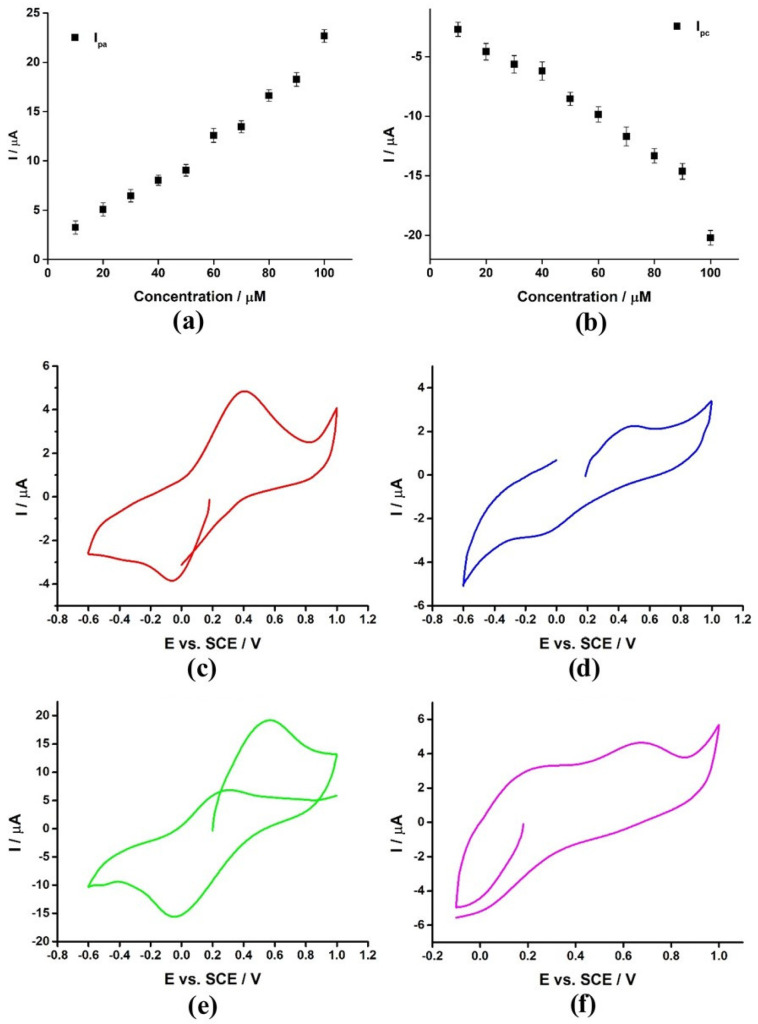
(**a**,**b**) Linear dependence of *I* vs. concentrations of NO_3_^−^ and (**c**–**f**) CV curves of GA@PAni-CNT at the different start and end potentials.

The adsorption of PAni on the electrode surface in the presence of a varying concentration of NO_3_^−^ is presented in Figure 5a,b. As already discussed, the amount of ES formed directly depends on the initial concentration of NO_3_^−^; therefore, by increasing the concentration of NO_3_^−^, more non-redox conversion of LS ↔ ES is possible, and upon applying redox potential, more ES ↔ PN redox transition occurs. The concentration of NO_3_^−^ is in the range of 10–100 µM with an interval of 10 µM. The CV curves were recorded at 100 mV/s in the potential window of −0.6–+1.0 V. The result shows both anodic and cathodic peak currents linearly increase with the increase in the concentration of NO_3_^−^, attributing to diffusion-controlled ES ↔ PN redox transition in the presence of NO_3_^−^. Thus, the suitability of the GA@PAni-CNT electrode for indirect determination of NO_3_^−^ has been successfully established.

### 3.9. On the Oxidation Mechanism of GA@PAni-CNT Electrode

The ES ↔ PN redox transition was more pronounced in the presence of NO_3_^−^. To authenticate this, the CV curves of the GA@PAni-CNT electrode were recorded by using different start and end potentials. Figure 5c shows the CV curve in 0.1 M pH 5 phosphate buffer electrolyte and 40 µM NO_3_^−^ at a scan rate of 20 mV/s. Initial (*E_i_*) and final (*E_f_*) potentials were set as open circuit voltage (OCV), whereas the first potential sweep (*E*_1_) was fixed at −0.6 and the second potential sweep (*E*_2_) in the reverse direction was at +1.0 V. At OCV, no potential or current was applied to the working electrode so the evolution of the redox transitions of the PAni could be performed. As is observable, clear redox transition appeared at 0.4/−0.05, which was due to the ES ↔ PN redox transition. In Figure 5d, *E_1_* and *E_2_* were set as +1.0 V and −0.6 V, respectively, while *E_i_* and *E_f_* remained at OCV. Even though the direction of sweep was reversed, the two redox peaks at +0.49 V and −0.057 V were a pair of ES ↔ PN. For the third curve in Figure 5e, *E_f_* changed to +1.0 V, whereas *E*_1_ and *E*_2_ were unchanged. Compared with Figure 5c, the redox peak currents of ES ↔ PN redox transition were considerably higher. Moreover, a new anodic peak associated with LS ↔ ES redox transition appeared at +0.263 V might be due to the adsorption of PAni on the electrode surface. In curve four in Figure 5f, it can be seen that by setting *E_f_*, *E*_1_, and *E*_2_ as −0.1 V, −0.1 V, and +1.0 V, respectively, the same anodic peak of LS ↔ ES transition emerges at +0.191 V and the anodic peak of ES ↔ PN redox transition dramatically shifts towards the more positive potential of +0.66 V. Interestingly, the anodic current of both the transitions were quite similar; however, no cathodic peak (or peaks) was seen in the reverse direction. Thus, by changing the start and end potentials, the two typical transitions of PAni were visible but suffered greater activity loss. In other words, NO_3_^−^ involvement in the non-redox transition of LS ↔ ES was minimized, which was not favorable for indirect electrochemical determination of NO_3_^−^. On the other hand, it was confirmed that the redox pair that occurs at ~0.32/0.039 was mainly due to the ES ↔ PN redox transition.

### 3.10. Selection and Optimization of SWV Response

**Figure 6:**
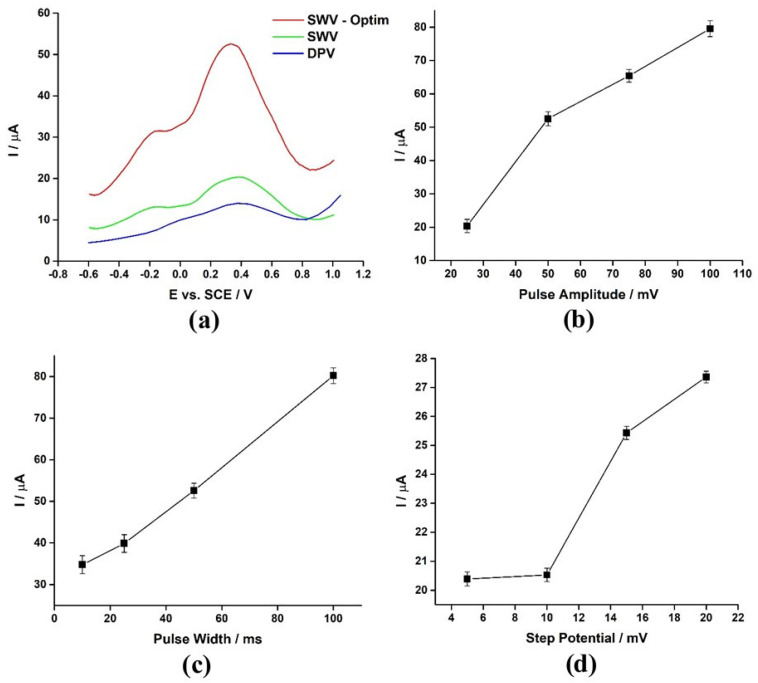
(**a**) Comparison of DPV, SWV, and SWV curves (under optimum conditions) and plots of *I* vs. (**b**) pulse amplitude, (**c**) pulse width, and (**d**) step potential.

The advantage of SWV over CV and differential pulse voltammetry (DPV), as the fastest scan rate technique, is obvious [69,85], because CV established that the adsorption of PAni at a lower scan rate can occur. Therefore, the use of SWV as a detection technique offers a greater advantage by generating a higher current of ES ↔ PN redox transition. In Figure 6a, the SWV and DPV curves obtained at the default parameter settings of the VSP-300 multichannel potentiostat are compared. Although the higher current density of the SWV curve than that of the DPV curve was understandable, in both the curves, the two redox transitions associated with the pairs of LS ↔ ES and ES ↔ PN are noticeable. This indicated that some amount of ES is generated by the redox transition of LS ↔ ES while performing SWV or DPV. The same will be discussed later. At this point, more emphasis was to obtain an optimized and higher SWV signal. The variation in the redox peak current of ES ↔ PN redox transition as a function of SWV pulse amplitude is demonstrated in Figure 6b, where a linear increase in the current until 50 mV followed by a slight decrease at higher amplitudes is observable. Under SWV pulse width influence (Figure 6c), a slow increase in the current from 10 to 25 ms and then a linear increase until 100 ms was observed. The dependence of ES ↔ PN redox peak current on the pulse step potential is depicted in Figure 6d, where a linear increase between 10–15 mV is observable. Although the current increased SWV peak shape was found to be sensitive to the change of the step potential, well-defined peak results using a step potential in the range of 5–10 mV were obtained. Therefore, using the optimized SWV parameters the improved SWV signal, which is displayed in Figure 6a, was also investigated in different buffer electrolytes.

### 3.11. Buffer Electrolyte Selection and pH Study

**Figure 7:**
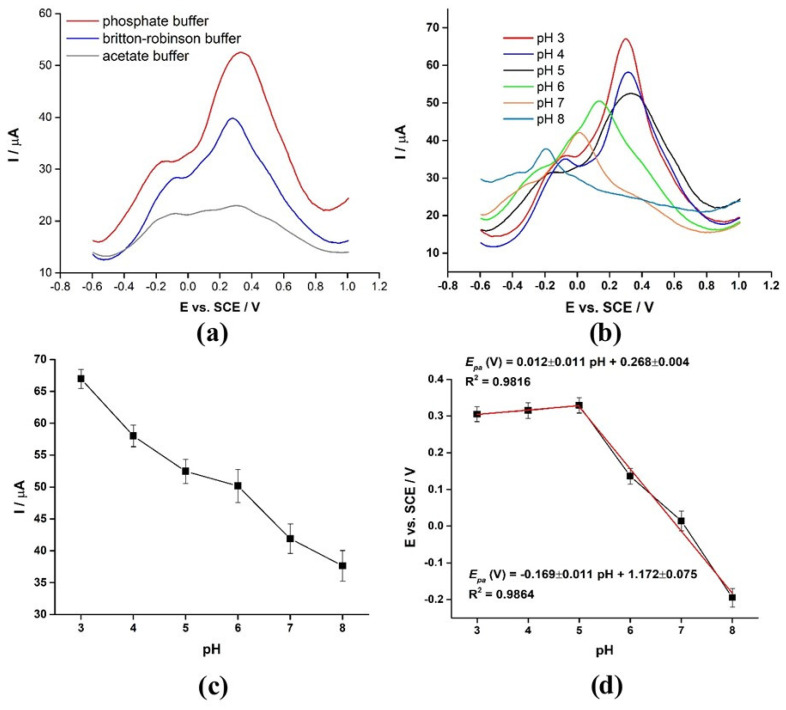
(**a**) Comparison of SWV curves in different buffer electrolytes, (**b**) SWV curves as a function of pH, and plots of (**c**) *I* vs. pH and (**d**) *E* vs. pH.

The influence of buffer solution on electrochemical signals was essential, as PAni protonation/deprotonation, which played a key role in forming a complex with NO_3_^−^, is dependent on buffer composition; therefore, the SWV curves of the GA@PAni-CNT electrode in three different buffer mediums 0 f 0.1 M concentrations and containing 40 µM NO_3_^−^ were recorded. As displayed in Figure 7a, regardless of whether Britton–Robinson buffer or acetate was used, both buffer solutions manifested inhibition effects on the SWV signal, suggesting the inappropriateness of both the buffer solutions while utilizing PAni or PAni-based electrodes for electrochemical sensing applications. The observed inhibition effects were assumed to be primarily due to the presence of acetic acid in both the buffers, as the interaction between polyaniline and acetic acid has been reported in the literature [86,87]. Because the GA@PAni-CNT electrode exhibited an enhanced SWV signal in phosphate buffer electrolyte, it was essential to examine the pH effect using the same buffer electrolyte system.

It was interesting to observe the effect of pH on the determination of NO_3_^−^ over the GA@PAni-CNT electrode. The SWV curves of GA@PAni-CNT electrode recorded as a function of pH in the range of 3–8 are shown in Figure 7b. Well-defined SWV curves were observed with pH values of 3, 4, and 5, which was in agreement with the literature [78,79], where electrostatic attraction and hydrogen bonding between NO_3_^−^ and LS were reported between pH 4 and 6. The potentials of the redox peaks shifted towards lower values with increases in pH over 5, while the peak currents and shape were drastically affected. A stronger influence of pH variation on the ES ↔ PN redox transition than on the LS ↔ ES redox transition has also been reported, which also agrees with our system [78,79]. The results indicated that both LS ↔ ES and ES ↔ PN redox transitions of GA@PAni-CNT electrode were more easily observed in the phosphate buffer electrolyte in the pH range of 3–5. A linear decrease in the peak current of ES ↔ PN redox transition with the increase in the pH. Figure 7c suggests that the redox activity loss of the GA@PAni-CNT electrode occurs at higher pH values. Moreover, the position of ES ↔ PN redox transition remained unaltered in the pH range of 3–5 but abruptly shifted towards lower values above pH 5 (Figure 7d), which was indicative of proton involvements in the redox transition of ES ↔ PN. Explicitly, the current measurement of ES ↔ PN redox transition at pH 3 was higher than other values; therefore, it was preferable to observe a linear dependence on the concentration of NO_3_^−^. In addition, as shown in Figure 7d, the observed two linear correlations of *E_pa_* (V) = 0.012 ± 0.011 pH + 0.268 ± 0.004 (R^2^ = 0.9816) and *E_pa_* (V) = −0.169 ± 0.011 pH + 1.172 ± 0.075 (R^2^ = 0.9864) in the pH ranges of 1–3 and 5–8, respectively, show slopes less than ~59 mV, which is quite indicative of the involvement of an unequal number of electrons and protons during the ES ↔ PN redox transition and supports the illustrated mechanism of NO_3_^−^ determination.

### 3.12. Analytical and Interference Concerns

**Figure 8:**
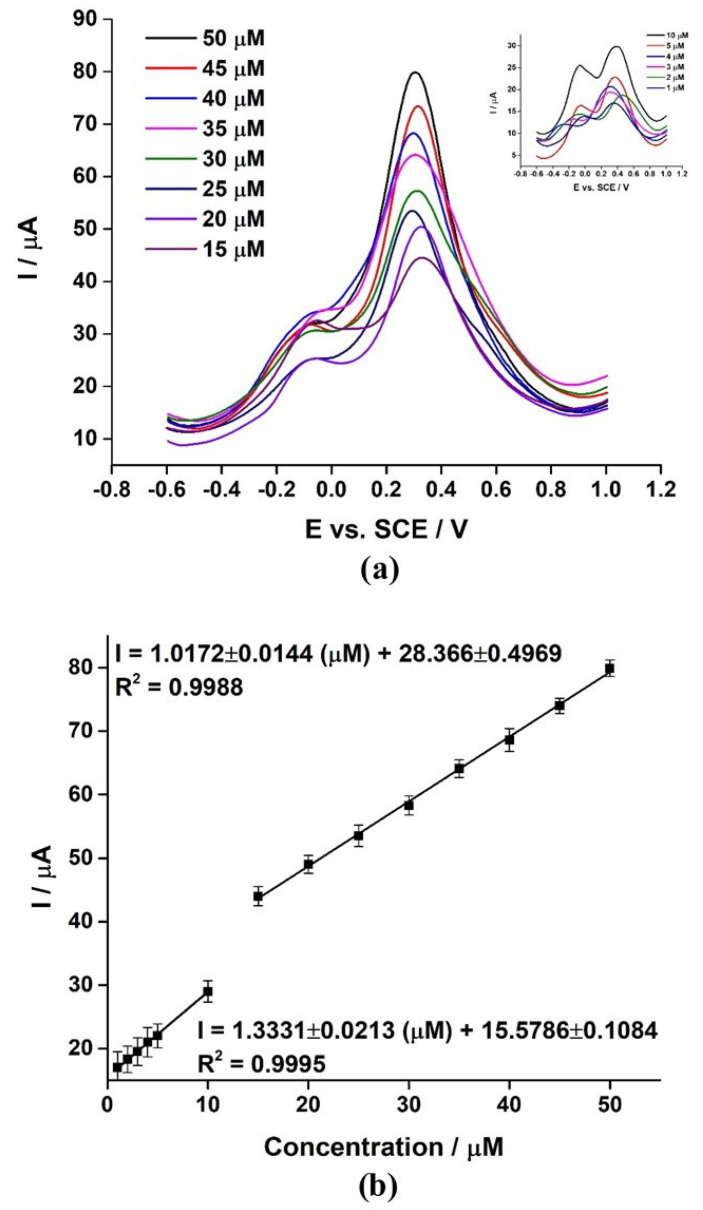
(**a**) SWV curves of GA@PAni-CNT in two different concentration ranges of NO_3_^−^ and corresponding (**b**) linear relationships.

Because the current generated by the redox pair of ES ↔ PN excluded the background in 0.1 M phosphate buffer electrolyte corresponded to the NO_3_^−^ concentration, NO_3_^−^ concentration-dependent SWV curves were recorded (Figure 8a). It is important to mention that the peak current of LS ↔ ES redox transitions is independent of the concentration of NO_3_^−^ and does not make any difference to the peak current of ES ↔ PN redox transition. Moreover, in the absence of NO_3_^−^ in the 0.1 M phosphate buffer electrolyte, the anodic peak of LS ↔ ES redox transition resulted in the same current as with 0.1 M phosphate buffer electrolyte and NO_3_^−^. Consequently, two linear dependence regions of NO_3_^−^ concentration covering the range 1–10 µM and 15–50 µM are reflected in Figure 8b. The two linear regression equations are *Ip_ES↔PN_* (µA) = 1.0172 (µM) + 28.366 (R^2^ = 0.9988) and *Ip_ES↔PN_* (µA) = 1.3331 (µM) + 15.5786 (R^2^ = 0.9995). The GA@PAni-CNT electrode shows a detection limit (3SD/b) of 0.42 µM at a signal-to-noise ratio of 3, which was quite promising and lower than the values reported so far (Table 1) for indirect electrochemical determination of NO_3_^−^ using modified zeolitic carbon paste electrode and Ag-doped zeolite-expanded graphite-epoxy electrodes [33,36,41,42,88]. The GA@PAni-CNT electrode illustrated a sensitivity of ~443 A/M cm^2^, obtained through the slope of the linear regression. The GA@PAni-CNT electrode reproducibility was of concern, which was less than 5% in terms of relative standard deviation for three repeated measurements.

Considering electrostatic interactions and hydrogen bonding between PAni and negative anions, interference in the determination of NO_3_^−^ was examined by adding Cl^−^, Br^−^, I^−^, SO_4_^−2^, NO_2_^−^, and CH_3_COO^−^ (Table 2). In the presence of each interfering agent separately, the deviation in the ES ↔ PN redox peak of more than ±5% was interpreted as interference. While the concentration of each interfering agent was the same as NO_3_^−^, the redox activity of ES ↔ PN transition was depressed severely only in the presence of CH_3_COO^−^. A weakened ES ↔ PN transition in acetic acid buffer electrolyte was determined to be present, which is consistent with the finding that the electrochemical determination of NO_3_^−^ using GA@PAni-CNT electrode in acetic acid (acetate ion) buffer should be avoided.

### 3.13. NO_3_^−^ Recovery

As summarized in Table 3, the prepared GA@PAni-CNT electrode showed better recovery of NO_3_^−^ in tap water samples when compared to most of the reported electrodes for the determination of NO_3_^−^. The result implied that the GA@PAni-CNT electrode can be successfully used for the electrochemical determination of NO_3_^−^ in real water samples.

## 4. Conclusions

The GA@PAni-CNT electrode was successfully prepared by catalytic polymerization of aniline in the presence of CNT and GA in order to investigate the indirect electrochemical determination of NO_3_^−^ in phosphate buffer electrolyte. The characterization verified that the GA@PAni-CNT electrode carried the leucoemeraldine form of PAni. The enhanced ES ↔ PN redox transition observed in the presence of NO_3_^−^ was actually due to the non-redox transition of LS ↔ ES by NO_3_^−^ complex formation. The study revealed a highly attractive option for indirect electrochemical determination of NO_3_^−^ using the GA@PAni-CNT electrode. Under the optimized SWV conditions, the GA@PAni-CNT electrode delivered a detection limit of 0.42 µM with high sensitivity, good reproducibility, negligible interference, and high recovery percentages of NO_3_^−^ in the tap water sample. The obtained results may open a compelling new avenue for the electrochemical determination of NO_3_^−^ in water, which is an imperative concern for environmental protection and public health.

## Figures and Tables

**Table 1:** Comparison of the analytical features of GA@PAni-CNT electrode with some of the reported works.

Electrode	Indirect/Direct Detection	Method	Linear Range (µM)	DL (µM)	Sensitivity (A/M cm^2^)	References
GA@PAni-CNT	Indirect	SWV	1–10 15–50	0.42	~443 A/M cm^2^	This work
Fe(II)-NClin/CPE	Indirect	SWV	80–1100	5.4	90 µA (µM)	[42]
SMDE	Indirect	SWP	0.6–5	2	-	[88]
AgZEGE	Direct	MPA	1000–10,000	80	0.0166 mA mM^−1^	[33]
AgZEGE	Direct	DPV	1000–10,000	500	2 × 10–3 mA mM^−1^	[41]

SMDE = static mercury drop electrode; SWP = square wave polarography; MPA = multi-pulsed amperometry.

**Table 2:** Deviation in the ES ↔ PN redox peak by negative anions.

Inferences	Concentration (µM)	*E*_ES↔PN_ Change (%)	*I*_ES↔PN_ Change (%)
Cl^−^	40	1.36	0.96
Br^−^	40	1.07	1.72
I^−^	40	0.89	1.86
SO_4_^−2^	40	1.10	0.72
NO_2_^−^	40	−2.35	4.34
CH3COO^−^	40	5.97	25.76

**Table 3:** Recovery results of NO_3_^−^ detection in the tap water sample.

Analyte	Detected	Added (µM)	Found (µM)	Recovery (%)
NO_3_^−^	0	20	19.43 ± 1.53	97.3 ± 2.61
	0	40	42.60 ± 1.10	106.5 ± 3.53
	0	60	60.09 ± 1.37	100.2 ± 2.83
	0	80	80.18 ± 0.99	100.2 ± 2.10

## Data Availability

Data will be made available on request.
